# Do plant traits predict the competitive abilities of closely related species?

**DOI:** 10.1093/aobpla/plv147

**Published:** 2015-12-31

**Authors:** Lauren M. Schwartz, David J. Gibson, Bryan G. Young

**Affiliations:** 1 Department of Plant Biology, Center for Ecology, Southern Illinois University, Carbondale, IL 62901, USA; 2 Department of Botany and Plant Pathology, Purdue University, West Lafayette, IN 47907, USA; 3 Present address: Department of Crop, Soil, and Environmental Sciences, University of Arkansas, Fayetteville, AR 72704, USA

**Keywords:** Amaranthaceae, competition, early vegetative growth, invasive species, resource drawdown

## Abstract

Invasive species are a threat to every ecosystem. There is a strong incentive to predict which species will become invasive before they become too widespread and unmanageable. Different approaches have been advocated to assess invasive species potential. These include examining plant functional traits, quantifying competitive ability and phylogenetic comparison. In this study, we conducted experiments based on the above approaches in a multi-year, temporally replicated, set of experiments to compare these assessment methods to determine the invasive potential of Japanese chaff flower (*Achyranthes japonica*). We compared plant traits and competitive ability of Japanese chaff flower with two agricultural invasive species, Palmer amaranth (*Amaranthus palmeri*) and tall waterhemp (*Amaranthus tuberculatus*), and one endangered plant species, bloodleaf (*Iresine rhizomatosa*), in the Amaranthaceae. Additionally, we assessed the invasive potential based on each of these approaches and determined the degree of agreement between them. A relatively conservative assessment integrating all three approaches would be that the competitive ability of closely related individuals with similar functional traits would share invasive potential. In a greenhouse experiment, each of the study species and soya beans were grown as monocultures and were evaluated to assess the drawdown of an aboveground (light) and a belowground (nitrogen) resource. In a field experiment, each study species was grown at varying densities per 15-cm-diameter pot with or without one or two soya bean plants, to simulate relative densities for soya beans grown in 38- and 76-cm-wide row spacing, respectively. In addition, Japanese chaff flower seedlings were planted either as un-manipulated seedlings or as a seedling cut back to the soil surface at the four-node stage (cut Japanese chaff flower) at which point seedlings have reached a perennial growth stage. The greenhouse experiment showed that each species drew down light differently, but not nitrogen. Shading decreased the aboveground biomass of the species in comparison with unshaded controls. Nitrogen, however, increased the aboveground biomass of Palmer amaranth and Japanese chaff flower. In the field experiment, a competitive effect ranking was determined to be: tall waterhemp ≥ Palmer amaranth = cut Japanese chaff flower ≥ uncut Japanese chaff flower ≥ bloodleaf, with the competitive response ranking being the inverse. These results suggest that under specific conditions, these closely related species do exhibit similar competitive abilities. Furthermore, the invasiveness and not the life history or habitat of these closely related species appeared to be the driving factor of competitiveness.

## Abstract (precis)

There is a strong incentive to predict which species will become invasive before they become too widespread and unmanageable. In this study, we conducted a multi-year, temporally replicated, greenhouse and field experiment based on plant functional traits, quantifying competitive ability, and phylogenetic comparison to determine the invasive potential of four closely related species varying in invasion status, life history and habitat. Our results suggest that these closely related species do exhibit similar competitive abilities and that the invasiveness and not the life history or habitat of these species appear to be the driving factor of competitiveness.

## Introduction

Invasive species have large ecological impacts on native species, communities and ecosystems (Elton 1958; Simberloff *et al*. 1997; Blackburn *et al*. 2014). There are ∼50 000 invasive species and the number is steadily increasing (Sakai *et al*. 2001). About 42 % of the species on the threatened and endangered species list are at risk primarily because of invasive species (Sakai *et al*. 2001; Pimentel *et al*. 2005). For example, in Illinois, the location of this study, 1156 invasive plant species had escaped cultivation and became naturalized by 2014, equivalent to 32.1 % of the state's total flora (Mohlenbrock 2014). Of that percentage, 78 % of the species were introduced from outside of North America (IDNR 1994). However, predicting whether or not an introduced species will become invasive can be difficult although there is a strong incentive to determining which plants are likely to become invasive before they become too widespread and unmanageable (Westbrooks 2004).

Different approaches have been advocated to assess invasive potential. These include examining plant functional traits, quantifying competitive ability and phylogenetic comparisons. Of these, ecologists are increasingly relying on plant functional traits as a way to understand some of the most fundamental and applied questions because trait-based approaches can help to disentangle the effect of ecological processes on communities (Díaz *et al*. 2007; Carmona *et al*. 2015). Furthermore, testing these methods are critical because it forms a pathway by which plant functional traits influence community assembly, the outcome of biological invasions and species diversity effects on ecosystem function (McGill *et al*. 2006; Kraft *et al*. 2015). Some of the literature connects plant functional traits to competitive outcomes. For example, species may differ in traits that influence their ability to draw down shared limiting resources or produce offspring, and the resulting average fitness differences may favour competitive exclusion (Tilman 1982; Chesson 2000). Competitive ability is further correlated with plant functional traits (Zhang and Lamb 2012) in each life stage (Wang *et al*. 2010).

Competitive ability can be compared between species in two ways: first, by assessing the competitive effect of plants or the ability to suppress other individuals, and secondly, by assessing the competitive response of plants or the ability to avoid being suppressed (Goldberg and Landa 1991; Violle *et al*. 2009; Zhang and Lamb 2012). Mechanistically, competition can be understood in terms of Tilman's resource ratio model that predicts that the growth rate of an individual is determined by the two resources at the lowest availability relative to the plant's requirement for all resources (Tilman 1982, 1987, 1997; Lehman and Tilman 2000). In general, the specific rate of biomass change of a species is based on the limiting resources in the environment (Tilman 1985; Krueger-Mangold *et al*. 2006). The growth of a plant would decrease in the presence of a neighbouring plant if these plants consumed the same limiting resources (Tilman 1988, 1997; Maron and Marler 2007). While Tilman's resource ratio model has been widely used in natural systems, it is less widely applied in crop systems although the model still applies (Zimdahl 2004; Miller *et al*. 2005).

The Amaranthaceae family contains important agricultural weeds, invasive exotics and rare native plants providing a useful system to test concepts related to competitiveness among closely related species. In the US Midwest region, Palmer amaranth (*Amaranthus palmeri*) and tall waterhemp (*Amaranthus tuberculatus*) have been acknowledged as two of the most problematic and widespread agricultural weeds (Bensch *et al*. 2003; Ward *et al*. 2013). These species have many characteristics that make them successful weeds, including the ability to grow 2–3 m in height (Horak and Loughin 2000; Trucco and Tranel 2011), a prolonged period of seed germination and seedling emergence late into the row-crop growing season (Hartzler *et al*. 1999). Competition of 8 plants m^−2^, starting at crop emergence, resulted in Palmer amaranth reducing soya bean yields by 78 % compared with 56 % for tall waterhemp (Bensch *et al*. 2003). Furthermore, soya bean yield was reduced by 10 % when plants emerged at the V4 growth stage of soya bean (Steckel and Sprague 2004; Steckel *et al*. 2004, 2008). Palmer amaranth and tall waterhemp have been found to be very competitive not only with row crops but also with other *Amaranthus* species (Trucco and Tranel 2011).

Japanese chaff flower (*Achyranthes japonica*) is also in the Amaranthaceae and represents a relatively recently introduced species that is spreading across the Ohio River Valley (Schwartz *et al*. 2015, 2016). This monoecious, perennial, C_3_ herb is native to Korea, China and Japan (Sage *et al*. 2007; Choi *et al*. 2010; Evans and Taylor 2011; Schwartz 2014). Japanese chaff flower is generally found in areas with some shade and moist soil. However, the species can also grow in drier areas in sun and in densely shaded areas (Schwartz 2014, 2015). Dense patches of Japanese chaff flower have been found in bottomland forests, riverbanks, field edges and in ditches and swales (Evans and Taylor 2011; Schwartz 2014; Schwartz *et al*. 2016). Large patches of Japanese chaff flower have shown indications of deer browsing and insect feeding, but the plant will release new shoot growth from previously dormant axillary buds and overcompensate (Schwartz *et al*. 2016). Apart from anecdotal observations, little has been reported on this species and only recently has an aggressive research campaign been launched to learn more about this species. The occurrence of Japanese chaff flower in row-crop field margins in southern Illinois has prompted concern about its potential competitive effects on crops. In contrast, bloodleaf (*Irisine rhizomatosa*) is classified as endangered in Illinois and Maryland and is considered to be rare in Indiana (IDNR 1994; Gibson and Schwartz 2014). Despite its endangered and rare status, very little ecological work has been conducted on this species (Gibson and Schwartz 2014).

In this study, we conducted experiments based on the above approaches in a multi-year, temporally replicated, set of experiments to compare these assessment methods to determine the invasive potential of Japanese chaff flower. We compared plant traits and competitive ability of Japanese chaff flower with two invasive agricultural species and one endangered confamilial plant species in the Amaranthaceae. Additionally, we assessed the invasive potential based on each of these approaches and determined the degree of agreement between them. Thus, the objective of this study was to determine the relative competitive effect and response of closely related species in the same family to a crop. Two hypotheses were tested: (1) the perennial species, Japanese chaff flower and bloodleaf, will have a lower resource requirement than the annual *Amaranthus* species when competing with interspecific neighbours (measured as resource drawdown) and (2) a competitive effect ranking would be Palmer amaranth > tall waterhemp > cut Japanese chaff flower = Japanese chaff flower = bloodleaf. The competitive response rankings will be inversely related among the four species. The rationale behind the hypothesized ranking was developed from personal observations and a literature review of the species.

## Methods

We tested our hypotheses by conducting two experiments. A resource drawdown experiment was conducted to test how each species utilizes an above- and belowground resource (test of Hypothesis 1), and a field experiment was conducted to determine the competitive effect and response of the study species on soya beans at varying densities and soya bean row spacing (test of Hypothesis 2).

### Resource drawdown experiment

The drawdown of light and soil nitrogen (N) by each species was determined in field soil under greenhouse conditions at the Southern Illinois University Tree Improvement Center (TIC) greenhouse. Seeds of each of the four Amaranthaceae species were collected from populations within 161 km of Carbondale, Illinois, each year. Japanese chaff flower seed was collected from Chestnut Hills Nature Preserve (CH: 37°11′N, 89°3′W) located in Pulaski county, Illinois, and bloodleaf seeds were collected from Beall Woods Nature Preserve (BW: 38°20′N, 87°49′W). Seeds of the two annual *Amaranthus* species (Palmer amaranth (located at the Belleville Research Center (BRC 9B: 38°30′N, 89°50′W)) and tall waterhemp (located at BRC T4: 38°31′N, 89°50′W) were collected from glyphosate susceptible populations and underwent a bleach (5.25 % sodium hypochlorite) scarification process to ensure maximum possible seed germination. The soya bean, Japanese chaff flower and bloodleaf seeds did not require pretreatment. Seeds of each species were sown into separate flats with potting soil and allowed to germinate. Seeding rates and timing were determined based on previously measured germination rates and establishment times for each species (e.g. Japanese chaff flower was seeded 10 days before tall waterhemp). When seedlings of each species had emerged, five seedlings per species were transplanted into each experimental pot. Seedling density was chosen based on pot size. We did not want the pot size to be a limitation to plant growth.

Field soil (0–15 cm depth) was collected from Southern Illinois University Agronomy Research Center (ARC). Soil was characterized as having a topsoil of silt loam (0–0.25 m) and subsoil (0.25–1.30 m) of silt clay loam (Herman *et al*. 1976). Field soil was sterilized and mixed in the ratio of 1 : 1 with sterilized sand to dilute the N concentration and aid in permeability while watering. The mixed soil was placed into 15-cm pots. The average greenhouse conditions included a photoperiod of ∼8–12 h per day, which were determined by supplemental lights in the greenhouse, and a temperature of 31 ± 5 °C. Two soya bean (Asgrow Brand AG3832 plot seed, Illinois origin) seeds were planted in each pot for a density equivalent to soya beans grown in a 38-cm row spacing in agricultural fields.

Resource manipulation treatments of N addition and light reduction (shading) were implemented. Nitrogen was added as granular ammonium nitrate applied at 1 g per pot prior to transplanting the seedlings. Shading treatments were implemented by surrounding the pots with a frame and then covering the frame with a 60 % shade cloth to simulate forested or crop canopies. A frame constructed of PVC pipe was placed around the non-shaded pots to control for shade effects produced by the frames. Pots were watered twice daily with 75 mL. There were five replicates (plus one unseeded control pot) of each treatment with two temporal replicates. Control pots were not sown with seeds to establish a baseline for resource drawdown values. Each run of the experiment lasted ∼4 weeks. Pots were placed in the greenhouse in a randomized complete block design.

#### Data collection

Light intensity drawdown was measured under the plants at the soil surface using a LI-COR Light Meter (Model LI-250; LI-COR, Lincoln, NE, USA) for each pot twice per week. Light quality was measured one time at the end of the experiment using an International Light 1400A radiometer/photometer (IL1400A; International Light, Inc., Newburyport, MA, USA) using white, blue, red (R) and far-red (FR) filters below the leaves. The light quality meter was placed below a leaf of each plant. Light quality was performed on a separate set of pots that did not undergo the N or light treatments.

Performance measurements (height and number of nodes) were recorded twice weekly to use as an indicator of early seedling growth. Above- and belowground biomass were harvested from each pot when the seedlings of each species had reached four nodes indicative of early seedling growth. Thus, all of the plants within a species were harvested at the same time, but the actual harvest date between species varied (i.e. the *Amaranthus* species reached the four-node stage before the perennial species and were harvested a week earlier). Biomass was oven dried (48 h, 55 °C) and weighed. Inorganic N was measured in the soil of each pot using ion-exchange resin bags (Binkley 1984). Resin bags were placed into the pot the day that the pots were sown and removed the day that the pots were harvested. Resin bags were constructed from nylon hose and consisted of 5 g of equal amounts of an anion (Dowex 1 × 8, 50–100 mesh; Acros Organics) and a cation (Dowex 50W × 8, 50–100 mesh; Acros Organics) resin. In the laboratory, the resin was extracted with 75 mL of 2 N KCl after shaking for 1 h at 200 r.p.m., filtered through a 0.4-µm filter membrane and the filtrate analysed for NH_4_-N and NO_3_-N on a Flow IV Solution Autoanalyzer (O.I. Corporation, College Station, TX, USA). Total N was determined by adding the NH_4_-N and NO_3_-N values.

### Seedling competitive effect and response experiment

#### Study site

Experimental plots were established at the Southern Illinois University, Carbondale TIC in Jackson County, Illinois (37°42′N, 89°16′W). The soil at the site was a silt clay loam, with a topsoil of silt loam and subsoil of silt clay loam (Herman *et al*. 1976). The experiment was conducted annually for 3 years (2012–14), with 2012 being a preliminary experiment (data not reported).

#### Experimental design

Seeds, which were collected in southern Illinois that year and were from the same seed source as the resource drawdown experiment, were planted in sterilized pots (15 cm diameter by 15 cm depth) filled with a silt clay loam soil collected from the TIC field. The soil was prepared as in the resource drawdown experiment. The soya bean, Japanese chaff flower and bloodleaf seeds did not require pretreatment. However, as in the resource drawdown experiment, the two *Amaranthus* species (Palmer amaranth and tall waterhemp) were scarified with bleach solution to promote germination. After seedling emergence, the seedlings were transferred to the field and the pots were submerged into excavated holes, so the soil surface in the field and pots were equivalent. Pots were used to prevent the release of Japanese chaff flower into the field, since at the time of this experiment this species had not been found in Jackson county, Illinois. In addition, the planting of an endangered species such as bloodleaf is heavily regulated and the pots provided containment. Volunteer plant seedlings were removed continually throughout the experiment. Each year (2012–14), the experiment was conducted until the plants within a species reached the end of the seedling stage (denoted by the majority of each species reaching the four-node stage) to seek consistent results. Thus, each species had one harvest, but the harvest date may have differed between species. Plants in this experiment were not grown beyond the seedling stage because control of agronomic weeds is frequently targeted at this stage.

Each of the four invasive species (*n* = 5 for invasive species treatment including cut and uncut Japanese chaff flower, see below) were planted either as a monoculture (control) or with soya bean (*n* = 2 for soya bean treatment) (Asgrow Brand AG3832 plot seed, Illinois origin). Japanese chaff flower seedlings were planted either as un-manipulated seedlings (referred to as uncut Japanese chaff flower, ACHJA) or as seedlings cut back to the soil surface at the four-node stage (cut Japanese chaff flower, ACHJA-C) at which point seedlings have reached a perennial growth stage (Smith 2013). The cut Japanese chaff flower plants represent perennial plants that may have survived the previous winter or regrowth from the application of a non-systemic herbicide typically applied prior to commercial soya bean planting. Upon emergence, the Amaranthaceae seedlings were thinned down to the desired seedling densities per pot (10, 30 and 90 for 38-cm rows (Trial 1) and 10 and 30 for 76-cm rows (Trial 2)). Each trial was conducted each year. From here forward, the 10 seedlings per pot density will be referred to as low, the 30 seedlings per pot as medium and the 90 seedlings per pot as high density. One or two equidistant soya bean seedlings were planted 4 cm apart in each pot to simulate typical planting densities of soya bean (Bensch *et al*. 2003) with the Amaranthaceae densities chosen to allow for agricultural conditions of crowding and competition around the soya bean plants. One soya bean per pot represented a 76-cm row spacing for soya beans (Trial 2), whereas two soya bean per pot represented a 38-cm row spacing (Trial 1).

This experimental design was an additive design with repeated measures (Gibson *et al*. 1999; Gibson 2015). The treatment design was a fully factorial combination of the four Amaranthaceae species including two stages of Japanese chaff flower (see below) (*n* = 5), four or three different densities (*n* = 4 (38-cm rows) or *n* = 3 (76-cm rows)), presence or absence of a soya bean cultivar (*n* = 2) and four or three replicates (*n* = 4 (38-cm rows) or *n* = 3 (76-cm rows)) for a total of 5 × 4 × 2 × 4 = 160 experimental units (pots) for the 38-cm rows and 5 × 3 × 2 × 3 = 90 experimental units (pots) for the 76-cm rows, a grand total of 250 pots (50 pots per species).

#### Data collection

Height (cm), number of branches, nodes and leaves were measured twice a week for each individual to determine performance. All seedlings in each pot were harvested when the majority of the individuals had reached the four-node stage, oven dried at 55 °C and both above- and belowground biomass weighed (g). Light intensity and soil moisture were measured twice per week in each pot using a LI-COR Light Meter (Model LI-250; LI-COR) and ECH_2_O Decagon Soil Moisture meter (Decagon Devices, Inc., Pullman, WA, USA), respectively.

### Statistical analysis

For the resource drawdown experiment, a three-way mixed model (SAS Institute) was used to determine the fixed effects of treatment (N, light), density and plant species on plant performance (plant height and nodes). Light quality was analysed using a two-way mixed model testing the effects of light wavelength and plant species on light reduction. The competition experiment was analysed using a repeated-measures three-way mixed model in SAS (PROC MIXED, SAS Institute) to detect treatment effects (fixed effect: weed density, soya bean presence or absence and weed species) on the performance (height, branch numbers, nodes and leaf numbers), light intensity and soil moisture. Random effects were blocks of pots in all tests. Aboveground and belowground biomass were analysed for the Amaranthaceae species and soya bean separately using a two-way mixed model to determine the effect of biomass and density or soya bean presence or absence. Significance was assessed at *P* < 0.05. A Tukey's test was used to determine significant differences among means with significant treatment effects. Based on weed species and soya bean performance, a competitive effect and response ranking was proposed (after Bensch *et al*. 2003; Zhang and Lamb 2012).

## Results

### Resource drawdown

**Figure 1.**
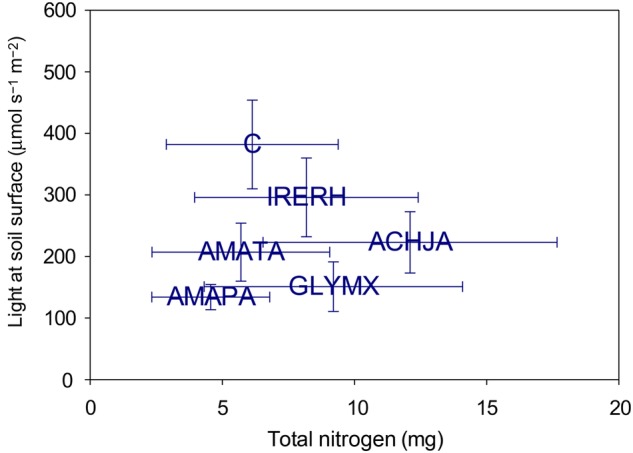
Relative resource drawdown for total N and light intensity at the soil surface for bloodleaf (IRERH), Japanese chaff flower (ACHJA), Palmer amaranth (AMAPA), tall waterhemp (AMATA), soya bean (GLYMX) and control (C) with 95 % confidence intervals. *n* = 40 plants/species.

In comparison with the controls (pots with no plants), the four Amaranthaceae species each drew down light, but not N (Fig. 1). In terms of light drawdown, bloodleaf drew down the least amount of light (which means that bloodleaf would have the highest percentage of light available at 69.2 ± 5.6 %), indicating that this species had the least amount of plant material shading the soil surface. Palmer amaranth and soya bean drew down the greatest amount of light (26.8 ± 3.5% and 30.2 ± 2.8 %, respectively), and tall waterhemp and Japanese chaff flower drew down an intermediate level of light in comparison with the other species (41.4 ± 2.9 and 45.6 ± 2.2 %, respectively).

**Figure 2.**
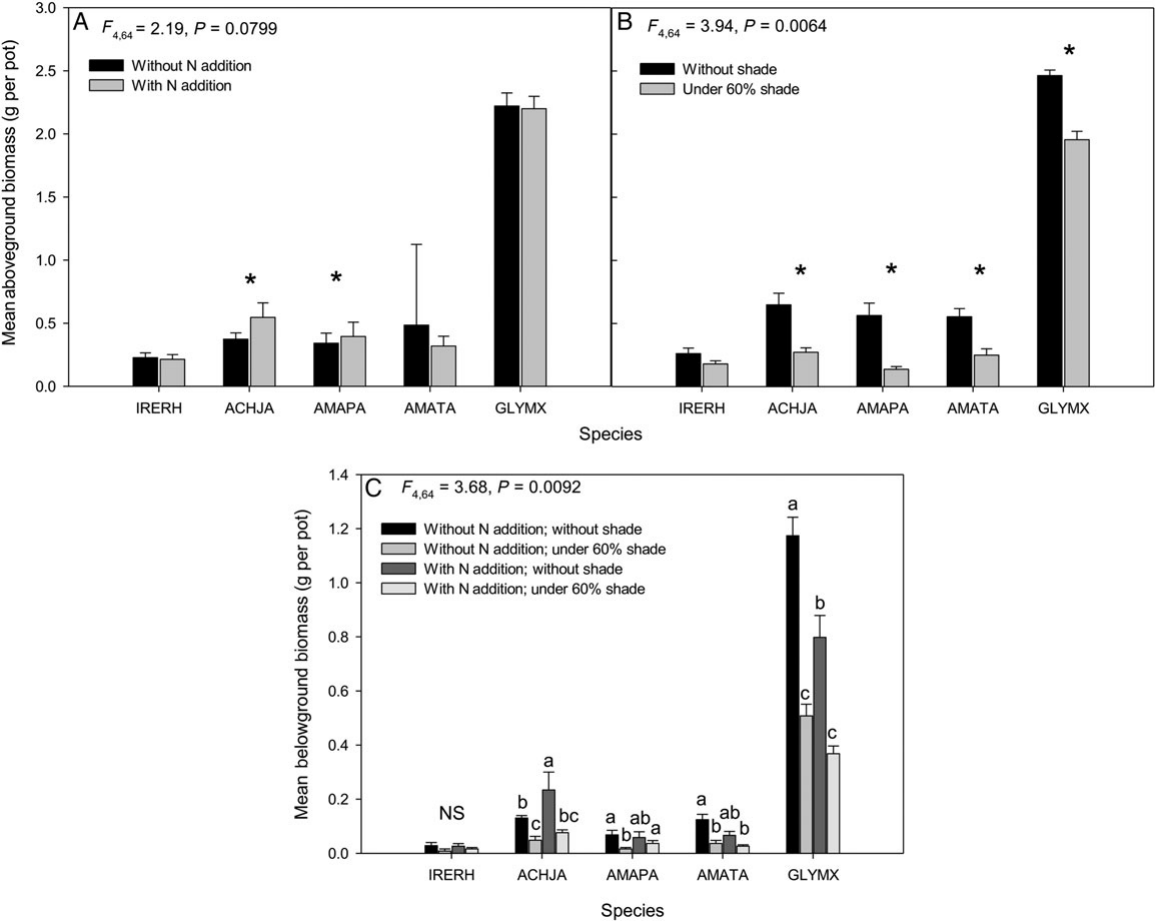
Mean (±SE) aboveground biomass in response to (A) N and (B) light treatments, and (C) belowground biomass in response to the interaction between N and light treatments. Species nomenclature is as follows: bloodleaf (IRERH), Japanese chaff flower (ACHJA), Palmer amaranth (AMAPA), tall waterhemp (AMATA) and soya bean (GLYMX). *Above pairs of bars indicates a significant difference (α = 0.05) between mean values in aboveground biomass comparisons between monocultures with or without N or light.

There was no significant difference for aboveground biomass between N treatment levels and species, except for Japanese chaff flower and Palmer amaranth (Table 1, Fig. 2A). A significant aboveground biomass interaction occurred between species and shading treatment (Fig. 2B). Each species produced more aboveground biomass without the shading than under the 60 % shading treatment, except bloodleaf. Again, Japanese chaff flower produced a similar amount of aboveground biomass (0.75 ± 0.03 g per pot) to both Palmer amaranth (0.6 ± 0.04 g) and tall waterhemp (0.55 ± 0.02 g) without shading. The soya bean crop produced the largest aboveground biomass (2.5 ± 0.03 g), whereas bloodleaf produced the smallest biomass (0.3 ± 0.01 g). Belowground biomass was affected by the shade treatment (Table 1), and there was a trend towards an increase for the *Amaranthus* species and a decrease for Japanese chaff flower and soya bean in belowground biomass with additional soil N (Fig. 2C). A greater amount of belowground biomass was attributed to the N addition for all species, especially Japanese chaff flower (2.7 ± 0.3 g). Belowground biomass of Palmer amaranth and tall waterhemp were similar regardless of soil N treatment without shading.

**Table 1.** *F* and *P* statistics for above- and belowground biomass (g), in the greenhouse experiment, for N and light for the four Amaranthaceae species and soya bean. df, degrees of freedom. ^1^Treatments with and without the 60 % shade cloth (*n* = 2). ^2^Treatments with and without the addition of ammonium nitrate (*n* = 2). ^3^All study species (*n* = 5).

Treatment/variable	df	Aboveground biomass		Belowground biomass	
		*F*	*P*	*F*	*P*
Shading^1^	1, 12	17	<0.0001	71.35	<0.0001
Nitrogen^2^	1, 12	17	<0.0001	61.05	<0.0001
Shading × nitrogen	4, 64	3.71	0.0864	9.39	0.0069
Species^3^	4, 28	59.9	<0.0001	19.89	<0.0001
Species × shading	4, 64	3.94	0.0064	6.26	0.0003
Species × nitrogen	4, 64	2.19	0.0799	4.12	0.0051
Species × shading × nitrogen	4, 64	59.9	0.6753	3.68	0.0092

**Figure 3.**
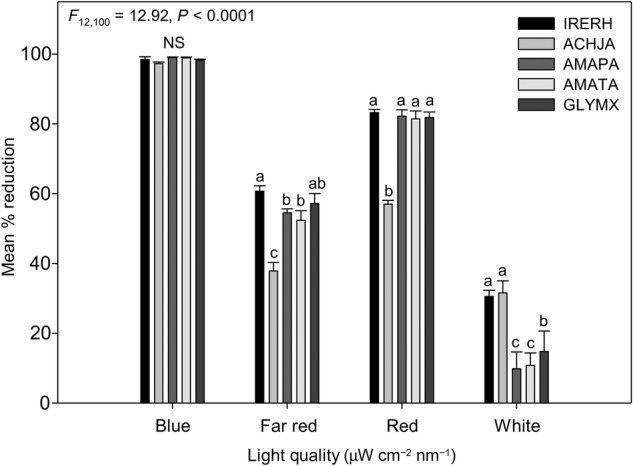
Mean (±SE) per cent reduction of light quality in response to study species and soya bean. Species nomenclature is as follows: bloodleaf (IRERH), Japanese chaff flower (ACHJA), Palmer amaranth (AMAPA), tall waterhemp (AMATA) and soya bean (GLYMX). Mean values with the same letters are not significantly different at *α* = 0.05 within a species.

An interaction between wavelength and species (*P* < 0.0001) was evident for the mean reduction in light quality (Fig. 3). Bloodleaf (60.7 ± 1.5 %) and soya beans (57.2 ± 2.9 %) had the lowest FR light reduction through shading, whereas Japanese chaff flower had the higher reduction (97.3 ± 0.5 %). The *Amaranthus* species had a similar reduction of FR light (AMAPA: 54.5 ± 1.1 %; AMATA: 52.4 ± 2.7 %). All of the species showed a mean per cent reduction in R light quality, but Japanese chaff flower reduced R light the least (57.0 ± 1.1 %). In this study, the R/FR ratios were comparable for all of the species (*P* > 0.05)—bloodleaf: 1.37 ± 0.58, Japanese chaff flower: 1.51 ± 0.45, Palmer amaranth: 1.51 ± 0.64, tall waterhemp: 1.56 ± 0.82 and soya bean: 1.43 ± 0.56.

### Competitive effect and response

**Figure 4.**
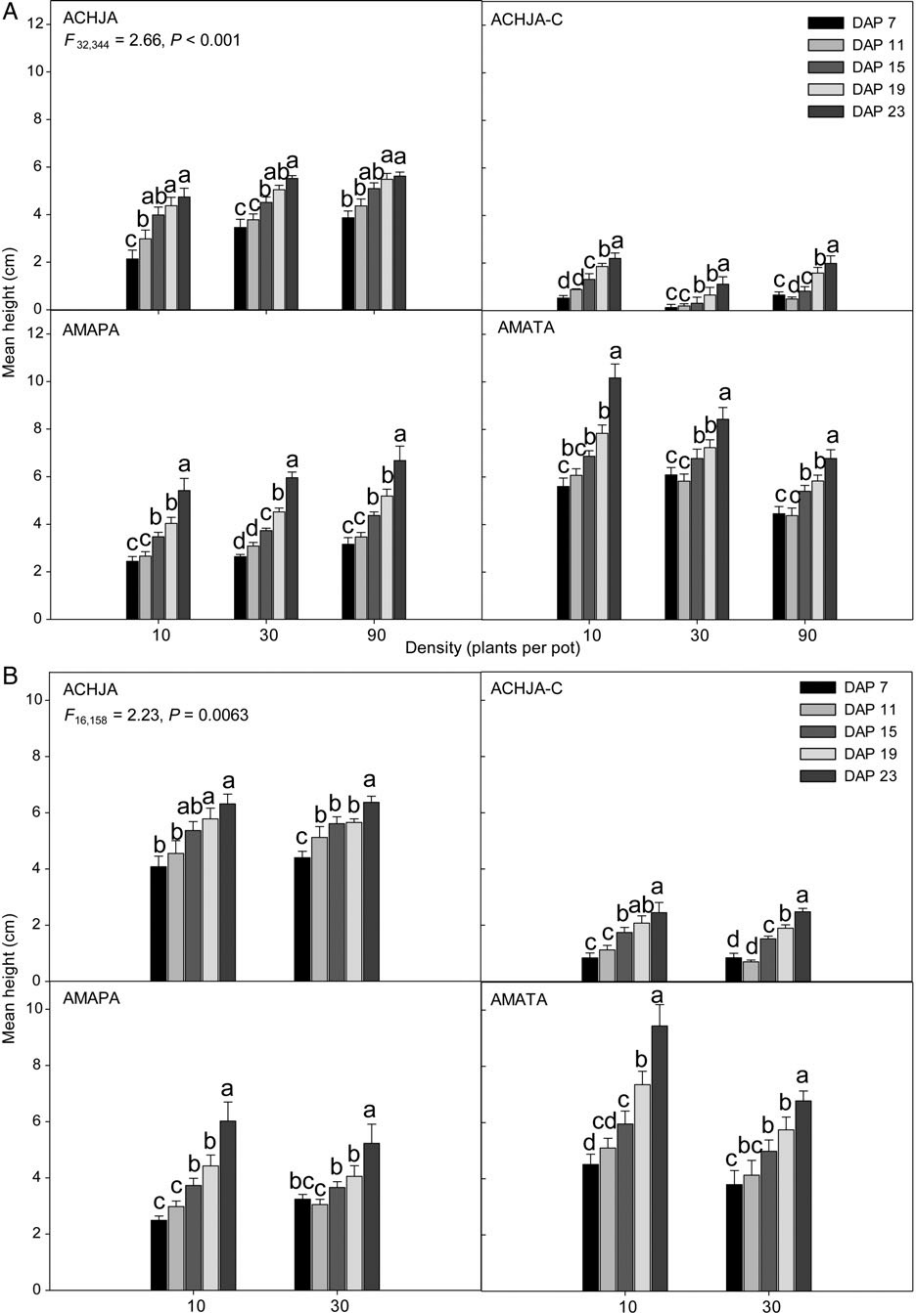

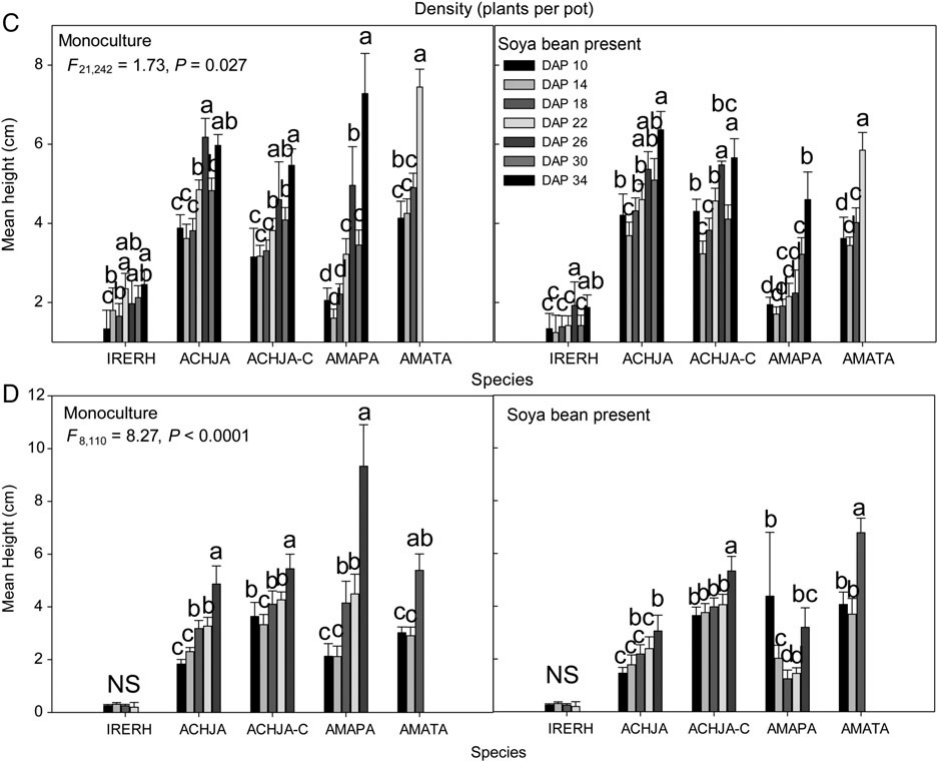
The competitive response of the mean (±SE) species height for (A) Trial 2 2013, (B) Trial 1 2013, (C) Trial 2 2014 and (D) Trial 1 2014 to soya bean. Study species nomenclature is as follows: bloodleaf (IRERH), uncut Japanese chaff flower (ACHJA), cut Japanese chaff flower (ACHJA-C), Palmer amaranth (AMAPA) and tall waterhemp (AMATA). Mean values with the same letters are not significantly different at *α* = 0.05 within a species.

The competitive response of the study species to soya bean was similar between trials within the same year. In 2013, for both trials, plant height was related to species, density and days after planting (Table 2). Tall waterhemp grew the tallest at the low and medium densities in both trials, with Palmer amaranth and uncut Japanese chaff flower grew to a similar height (Fig. 4A and B). Both Palmer amaranth and uncut Japanese chaff flower were not affected by density, whereas tall waterhemp was density sensitive. The cut Japanese chaff flower plants were the shortest regardless of trial. In Trial 1, at the highest density (90 seedlings per pot), both *Amaranthus* species reached the same height by the final day after planting (DAP) (Fig. 4A). In 2014, however, there was an interaction between species, DAP and soya bean (Table 2). Regardless of the trial, when soya beans were present, the height of bloodleaf was reduced (Fig. 4C and D). By DAP 23 in both trials, monocultures of seedlings of the two *Amaranthus* species were the largest, with both cut and uncut Japanese chaff flower seedlings only 1 cm shorter. Bloodleaf was the shortest in both trials each year. The competitive response between years, regardless of trial, was comparable with each other.

**Table 2.** Significant effects and interactions among groups based on Amaranthaceae species competitive effect and response (field experiment) to soya bean presence/absence. Only significant differences are shown within a variable. Pooled over species. *N*, the number of groups in a treatment or variable; df, degrees of freedom; T1, Trial 1; T2, Trial 2; Species, study species; Soya bean, soya bean(s) present; DAP, day after planting; Density, study species density (T1: 10, 30, 90; T2: 10, 30).

Variable/effect	*N*	df	*F*	*P*
Soil moisture				
T1-2013: Species × soya bean × DAP	60	8, 110	1.76	0.0366
T1-2013: Density × soya bean × DAP	36	8, 240	4.66	<0.0001
T1-2014: Soya bean × DAP	12	3, 147	7.77	0.0438
T2-2013: Species × DAP	30	15, 482	2.88	<0.0001
T2-2013: Density × soya bean × DAP	48	15, 588	3.89	<0.0001
T2-2013: Species × density × soya bean	40	16, 246	1.92	0.0034
T2-2014: Species × DAP	30	15, 588	2.19	0.0062
T2-2014: Soya bean × DAP	12	3, 322	5.33	0.0013
Light intensity at the soil surface				
T1-2013: Density × soya bean	8	2, 114	0.88	0.04184
T1-2014: Density × soya bean	6	2, 12.1	4.75	0.0299
T2-2013: Density × soya bean	8	3, 224	2.94	0.0338
T2-2014: Density × soya bean	6	3, 27.2	7.74	0.0007
Species height				
T1-2013: Density × soya bean	6	5, 90.9	4.62	0.0379
T1-2013: Species × density × DAP	90	16, 158	2.23	0.0063
T1-2013: Species × DAP × soya bean	60	8, 110	2.94	0.0003
T1-2014: Species × DAP × soya bean	60	8, 110	8.27	<0.0001
T1-2014: Species × density × DAP	90	16, 158	3.52	0.0012
T2-2013: Species × density × DAP	120	32, 344	2.66	<0.0001
T2-2014: Species × DAP × SB	60	21, 242	1.73	0.027
Soya bean height				
T1-2013: Species × density	15	8, 24.1	3.09	0.0151
T1-2014: Species × density	15	12, 64.1	1.94	0.0345
T2-2013: DAP × density	24	12, 229	5.06	<0.0001
T2-2013: Species	5	4, 68	2.92	0.0305
T2-2014: DAP	6	5, 78.3	31.88	<0.0001
T2-2014: Species × density	20	12, 64.1	1.98	0.0406
Species aboveground biomass				
T1-2013: Species × density	15	12, 82	3.37	0.0186
T1-2014: Species × soya bean	10	4, 30	4.92	0.0067
T2-2013: Species × density × soya bean	40	16, 138	2.66	0.0016
T2-2014: Species × soya bean	10	4, 66.9	5.02	0.0013
Soya bean aboveground biomass				
T1-2013: Density	3	2, 16	6.40	0.0051
T1-2014: Species	5	4, 86	15.01	<0.0001
T2-2013: Species × density	20	12, 49.3	1.79	0.0757
T2-2014: Species × density	20	12, 48	2.49	0.0127
Belowground biomass				
T1-2013: Species × density	15	12, 102	3.53	0.0026
T1-2014: Species × density × soya bean	30	8, 76.5	3.10	0.0361
T2-2013: Species × density	15	12, 102	3.35	0.0004
T2-2014: Species × density × soya bean	30	8, 76.5	3.75	0.001

**Figure 5.**
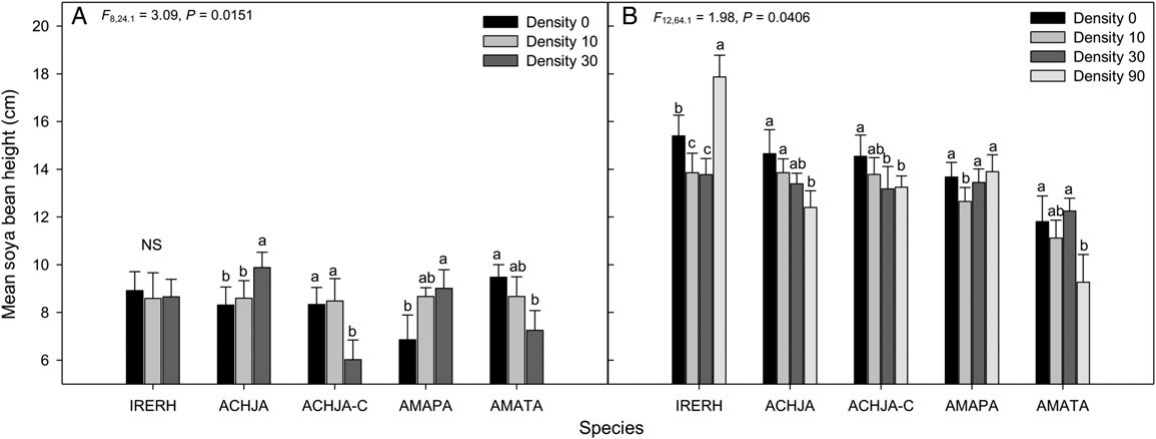
Competitive effect of mean (±SE) soya bean height for (A) Trial 1 2013 and (B) Trial 2 2014 in response to the species bloodleaf (IRERH), uncut Japanese chaff flower (ACHJA), cut Japanese chaff flower (ACHJA-C), Palmer amaranth (AMAPA) and tall waterhemp (AMATA). Mean values with the same letters are not significantly different at *α* = 0.05 within a species.

The competitive effect of the study species on soya beans was only apparent in Trial 2 in 2013 (Fig. 5A) and Trial 1 in 2014 (Fig. 5B). There was an interaction between species and density in both trials. In Trial 2 in 2013 (*P* = 0.015), the highest density of cut Japanese chaff flower reduced the height of soya bean the most, followed by the two *Amaranthus* species, uncut Japanese chaff flower and bloodleaf. For the lowest density, the cut Japanese chaff flower again reduced the height of the soya beans the most, followed by uncut Japanese chaff flower and Palmer amaranth. Although the reduction in height was relatively small (1.8–3.1 cm), both uncut Japanese chaff flower and the cut Japanese chaff flower reduced the height of soya bean in a similar manner to the two *Amaranthus* species with bloodleaf having no effect at all three densities. The same trend in soya bean height reduction across all densities occurred in Trial 1 in 2014: the presence of tall waterhemp caused the greatest height reduction, followed by the cut Japanese chaff flower and uncut Japanese chaff flower, with Palmer amaranth reducing the height the least. Consistency in results between trials and years supports intrinsic differences among species rather than short-term environmental variability (phenotypic plasticity).

There was an interaction between DAP, density and soya bean (Table 2) affecting soil moisture in 2013. The soil moisture in the pots with densities of 10, 30 or 90 seedlings per pot was relatively similar regardless of trial **[see** **Supporting Information—Fig. S1****]**. In 2014, however, there was an interaction between DAP and soya bean. In both trials at DAP 10, the monocultures had a lower mean soil moisture than soya bean (19.8 ± 3.5 and 28 ± 4.1 %, respectively), but on all other consecutive DAPs, the opposite was apparent. Mean light intensity at the soil surface for all years and trials, except Trial 2 in 2013 **[see** **Supporting Information—Fig. S2****]**, had an interaction between density and soya bean. Overall, light intensity at the soil surface decreased by 25 % in 2013 and 70 % in 2014 with an increase in the density **[see** **Supporting Information—Fig. S2****]**.

**Figure 6.**
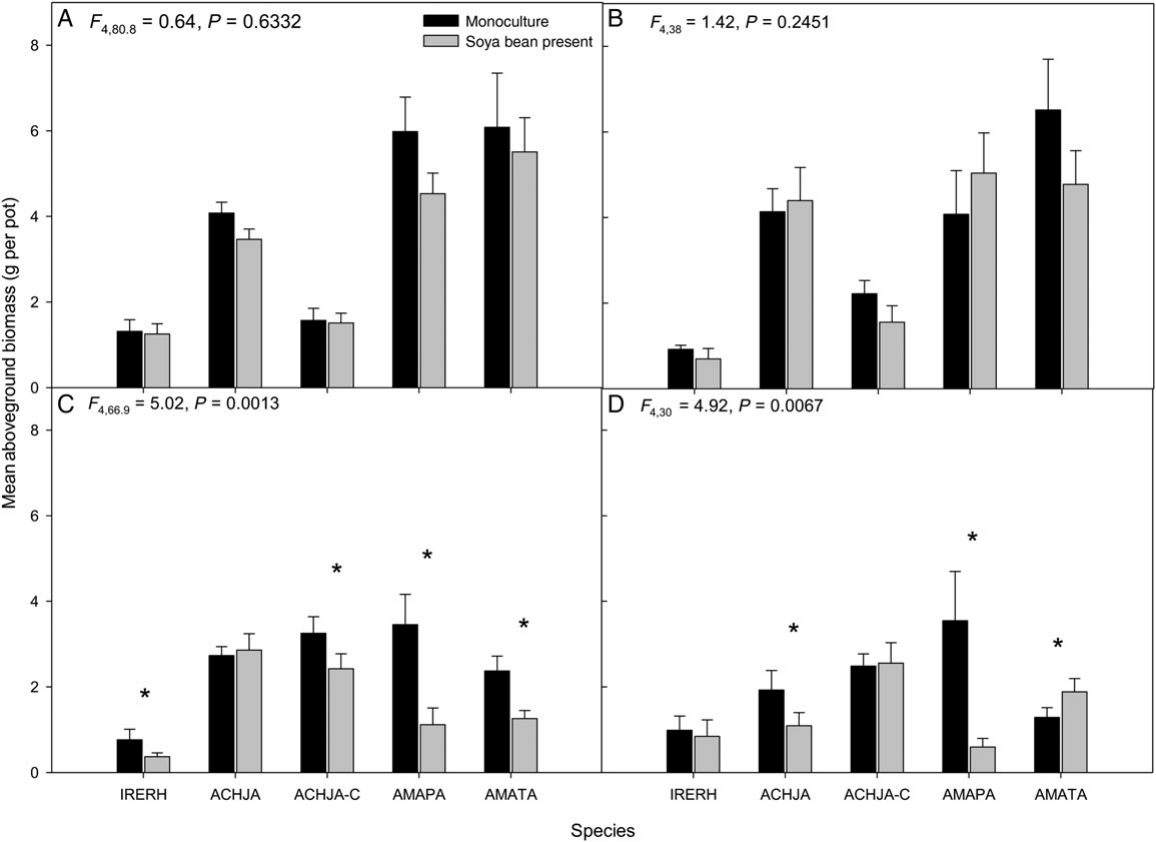
Mean (±SE) aboveground biomass for the study species bloodleaf (IRERH), uncut Japanese chaff flower (ACHJA), cut Japanese chaff flower (ACHJA-C), Palmer amaranth (AMAPA) and tall waterhemp (AMATA) in response to soya bean for (A) Trial 2 2013, (B) Trial 1 2013, (C) Trial 2 2014 and (D) Trial 1 2014. *Above pairs of bars indicates a significant difference (α = 0.05) between mean values in aboveground biomass comparisons between monocultures and in the presence of soybean.

Aboveground biomass was affected by study species and soya bean presence in the 2014 trials (Table 2, Fig. 6C and D) but not the 2013 trials (Fig. 6A and B). In both 2014 trials, the study species monocultures generally had a greater biomass than the mixtures with soya bean. Among the study species monocultures, Palmer amaranth had the greatest biomass (3.7 ± 0.7 g per pot). The cut Japanese chaff flower (Trial 1: 3.4 ± 0.6 g) and uncut Japanese chaff flower (Trial 2: 2.5 ± 0.4 g) had the next largest biomass. In Trial 2, tall waterhemp showed the opposite effect with greater biomass when soya bean was present. Data on the number of branches, nodes and leaves are not reported since these variables showed similar results to height (see Schwartz *et al*. 2016).

**Figure 7.**
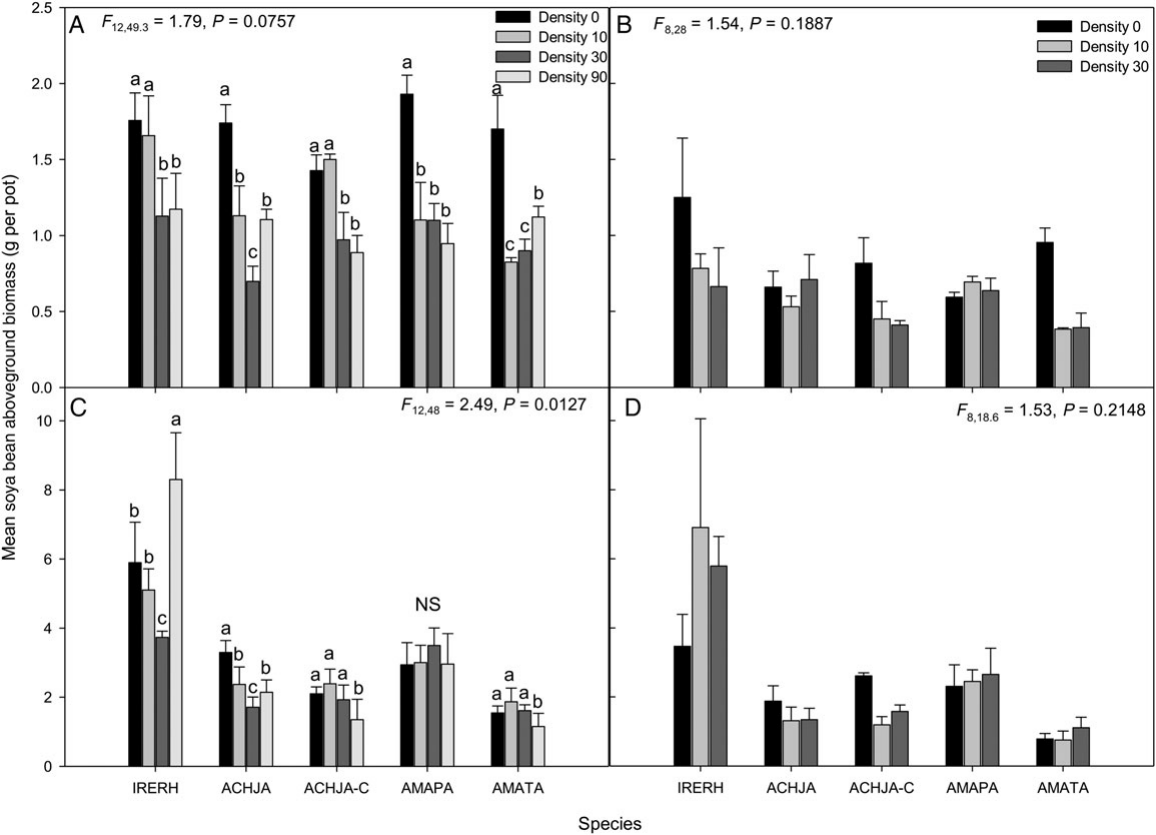
Mean (±SE) aboveground biomass for the soya bean in response to the species bloodleaf (IRERH), uncut Japanese chaff flower (ACHJA), cut Japanese chaff flower (ACHJA-C), Palmer amaranth (AMAPA) and tall waterhemp (AMATA) for (A) Trial 2 2013, (B) Trial 1 2013, (C) Trial 2 2014 and (D) Trial 1 2014. Mean values with the same letters are not significantly different at *α* = 0.05 within a species.

Neighbour species identity had a direct effect on soya bean biomass (Table 2). Aboveground biomass of soya bean was affected by the interaction between study species and density in only 2014 (2013: *P* = NS; 2014: *P* = 0.01). Regardless of year and density, the highest soya bean biomass was in the presence of bloodleaf indicating that it affected soya bean the least of the species (Fig. 7A and C). In 2013, the ranking of study species effects on soya bean varied with density (Fig. 7A).

An overall competitive effect and response ranking among the study species was developed from the results of both the greenhouse and the field experiment based on resource drawdown and competitive abilities. The competitive effect ranking was determined to be: tall waterhemp ≥ Palmer amaranth = cut Japanese chaff flower ≥ uncut Japanese chaff flower > bloodleaf. The competitive response ranking was the inverse.

## Discussion

In this study, we examined plant functional traits, competitive ability and phylogenetic relatedness of four closely related Amaranthaceae species. We assessed the invasive potential based on each of these approaches and determined the degree of agreement between them. Specifically, we considered resource drawdown as a functional trait and competitive effect and response to the soya bean crop to reflect competitive ability. A relatively conservative assessment integrating all three approaches would be that the competitive ability of closely related individuals with similar functional traits would share invasive potential. From this perspective, there are indications that Japanese chaff flower has high invasive potential. Thus, we advocate a multimodal approach to assessing invasive potential because each approach offers a different and complimentary dimension of information.

### Resource drawdown

The observed variation in resource drawdown among the four species can be explained in part by R* theory. An R* value simply is the concentration of a resource that a species requires to survive (Krueger-Mangold *et al*. 2006). The species with the lowest R* value will outcompete a species with a higher R* for that particular resource (Tilman 1982, 1988). Under the conditions of this experiment, seedlings of Japanese chaff flower and Palmer amaranth drew down the limiting resources in a similar manner, which indicates that at the early growth stage tested in this experiment, Japanese chaff flower could potentially affect a crop (i.e. soya beans) in a similar way as Palmer amaranth. Thus, these species would likely displace a species such as bloodleaf that show low rates of resource drawdown when grown in mixture. Although bloodleaf was not grown in conditions that were representative of its native environment (i.e. sterilized soil with fertilizer and a crop versus under a forest canopy), the low drawdown of light by bloodleaf may contribute to the slow growth of this perennial species with a poor ability to colonize. These characteristics coupled with habitat loss may have contributed to its endangered status in Illinois, Maryland and Indiana (Gibson and Schwartz 2014). Furthermore, the lack of differences between the species for N drawdown may also have been due to the short time frame of the experiments necessary to investigate early seedling competition and because resin bags average nutrient levels over the course of the trial (Klingaman and Oliver 1994). We hypothesize that if the experiment had run longer with plants growing beyond the seedling stage, that species differences would have been seen.

Ballaré *et al*. (1987) proposed that the FR wavelength was reflected by nearby leaves, which allowed for an early detection of neighbouring species that signalled oncoming competition during canopy development. Thus, as the seedlings in this study were growing, FR reflection among neighbours could have been signalling competition and initiating competitive responses through the FR/R phytochrome photoreceptor (Holt 1995). Novoplansky (1991) demonstrated *Portulaca oleracea* L. seedlings avoiding growth in the direction of species with higher reflected FR light. Thus, Japanese chaff flower with the lowest reduction in FR light among the species tested here may inhibit neighbouring species growing towards it. Plants growing in the shade of neighbouring taller vegetation are usually receiving reduced light intensity with a decreased R/FR ratio (Yang *et al*. 2014). Thus, plants grown under such conditions exhibit shade avoidance responses (i.e. elongated stem growth and little new leaf growth) (Smith 2000). Similar responses to decreased light intensity during growth have been reported for Palmer amaranth, where plasticity in acclimation to changing light conditions has enabled Palmer amaranth to develop in shade regions (i.e. under a crop canopy) and to achieve high rates of growth if suddenly exposed to high light (Patterson 1985).

### Competitive effect and response

The competitive effect/response ranking in this study is novel because the species that are being compared are within the same plant family, are found in different habitats and their competitiveness varies. In addition, competitive abilities have been based off of more than one trait (Andrew *et al*. 2015). Japanese chaff flower attained a high competitive ranking in this study, despite not being recognized as an agricultural weed. Rankings based on competitive abilities have been used in several other studies that range from closely related weeds (Andrew *et al*. 2015), to less closely related weeds (Horak and Loughin 2000; Bensch *et al*. 2003; Hock *et al*. 2006), or to cultivars of a single weed (Hansen *et al*. 2008; Andrew *et al*. 2015). Although Japanese chaff flower may not be fully suited to be the newest weed species in agriculture by escaping management strategies implemented by farmers (e.g. current susceptibility of Japanese chaff flower to herbicide modes of action limits its spread in agricultural systems; Smith 2013; Schwartz *et al*. 2016), it is still an aggressive weed that farmers and land owners need to be able to identify. This species has many similar characteristics to the *Amaranthus* species, such as the ability to colonize in areas with limiting resources, continual flushes of germination throughout the growing season, the ability to outcompete other weed species and high fecundity, but Japanese chaff flower also is a perennial species that can withstand removal of shoot material and has a high germination rate (Schwartz *et al*. 2016). Only early detection and rapid response methods can be relied on to keep Japanese chaff flower out of areas in and around agricultural fields. If this species evolves resistance to various herbicide modes of action, as have other taxa in the Amaranthaceae (Heap 2014), it may well become a prominent weed in agriculture. Indeed, the congener *Achyranthes aspera* L. infests field and vegetable crop fields carrying with it parasitic nematodes (Anwar *et al*. 2009).

Additionally, the environment plays a large role in the competitive effect/response of plant species. The similarity in competitiveness between years could be due, in part, to the very similar environmental factors during the month of May, when both trials were initiated. The precipitation levels did vary with 9 cm of precipitation in May in 2013 and 12.5 cm in 2014. Temperature is also an important ecological factor in determining species growth and productivity. Palmer amaranth and tall waterhemp exhibit their highest germination rate of 30 and 50 %, respectively, when mean air temperatures are at 25 °C (Guo and Al-Khatib 2003).

## Conclusions

This research serves as an indication that the functional traits conferring competitiveness of closely related species can be very similar, especially when comparing between invasion status (Garnier and Navas 2012). Although the invasive species of this study, Palmer amaranth, waterhemp and Japanese chaff flower, all exhibited similar competitive and general life-history traits to one another, their habitats do not overlap much in nature. Our study indicates that if these species shared the same habitat, then the perennial Japanese chaff flower could be just as competitive, if not more so, than the well-known annual *Amaranthus* species. Furthermore, as a species actively expanding its invasive range, Japanese chaff flower can potentially invade other habitats, such as agricultural or open fields, given the right conditions. A Japanese chaff flower invasion into agriculture fields is currently improbable. However, pre-existing evolutionary traits as seen in other Amaranthaceae species, e.g. to develop herbicide resistance (Vencill *et al*. 2008), is an evolutionary stepping stone for this species. Undoubtedly, specific management tactics implemented by individual growers or managers will have a significant influence on the rate that herbicide resistance could occur for Japanese chaff flower, as observed in other species (e.g. Beckie *et al*. 2004; Evans *et al*. 2015).

Overall, this study determined that competition between closely related species is driven by invasiveness and not by life-history traits. A confamilial comparison indicated that competitiveness of the perennial Japanese chaff flower related more towards the other invasive *Amaranthus* species than to the endangered native, perennial bloodleaf. Resource drawdown and competition with soya bean was comparable between all of the invasive species, which should raise concern for other invasive species in different habitats in the same geographic area. More research is needed, however, to determine whether the *Amaranthus* species would compete in a similar manner to Japanese chaff flower in a forested area and whether Japanese chaff flower would compete in a soya bean field as it did in this experiment.

## Sources of Funding

Partial funding was provided by the Illinois Department of Natural Resources (Grant Agreement No. 13-026W) to work on bloodleaf.

## Contributions by the Authors

All listed authors wrote the manuscript and designed the experiments. L.M.S. collected and analysed the data.

## Conflict of Interest Statement

None declared.

## Supporting Information

The following additional information is available in the online version of this article –

**Figure S1.** Mean (±SE) soil moisture for (A) Trial 2 2013, (B) Trial 1 2013, (C) Trial 2 2014 and (D) Trial 1 2014. Red lines are indicative of daily average soil moisture. Mean values with the same letters are not significantly different at *α* = 0.05 within a species.

**Figure S2.** Mean (±SE) light intensity at the soil surface for (A) Trial 2 2013, (B) Trial 1 2013, (C) Trial 2 2014 and (D) Trial 1 2014.

Additional Information

## References

[PLV147C1] Andrew IKS, Storkey J, Sparkes DL 2015 A review of the potential for competitive cereal cultivars as a tool in integrated weed management. Weed Research 55:239–248. 10.1111/wre.12137 27478257 PMC4950144

[PLV147C2] Anwar SA, Amjad Z, Nazir J 2009 *Meloidogyne incognita* infection of five weed genotypes. Pakistan Journal of Zoology 41:95–100.

[PLV147C3] Ballaré CL, Sánchez RA, Scopel AL, Casal JJ, Ghersa GM 1987 Early detection of neighbour plants by phytochrome perception of spectral changes in reflected sunlight. Plant, Cell and Environment 10:551–557.

[PLV147C4] Beckie HJ, Hall LM, Meers S, Laslo JJ, Stevenson FC 2004 Management practices influencing herbicide resistance in wild oat. Weed Technology 18:853–859. 10.1614/WT-03-124R

[PLV147C5] Bensch CN, Horak MJ, Peterson D 2003 Interference of redroot pigweed (*Amaranthus retroflexus*), Palmer amaranth (*A. Palmeri*), and common waterhemp (*A. rudis*) in soybean. Weed Science 51:37–43. 10.1614/0043-1745(2003)051[0037:IORPAR]2.0.CO;2

[PLV147C6] Binkley D 1984 Ion exchange resin bags: factors affecting estimates of nitrogen availability. Soil Science Society of America Journal 48:1181–1184. 10.2136/sssaj1984.03615995004800050046x

[PLV147C7] Blackburn TM, Essl F, Evans T, Hulme PE, Jeschke JM, Kühn I, Kumschick S, Marková Z, Mrugała A, Nentwig W, Pergl J, Pyšek P, Rabitsch W, Ricciardi A, Richardson DM, Sendek A, Vilà M, Wilson JRU, Winter M, Genovesi P, Bacher S 2014 A unified classification of alien species based on the magnitude of their environmental impacts. PLoS Biology 12:e1001850 10.1371/journal.pbio.1001850 24802715 PMC4011680

[PLV147C8] Carmona CP, Rota C, Azcárate FM, Peco B 2015 More for less: sampling strategies of plant functional traits across local environmental gradients. Functional Ecology 29:579–588. 10.1111/1365-2435.12366

[PLV147C9] Chesson P 2000 Mechanisms of maintenance of species diversity. Annual Review of Ecology and Systematics 31:343–366. 10.1146/annurev.ecolsys.31.1.343

[PLV147C10] Choi CY, Nam HY, Chae HY 2010 Exotic seeds on the feathers of migratory birds on a stopover island in Korea. Journal of Ecology and Field Biology 33:19–22. 10.5141/JEFB.2010.33.1.019

[PLV147C11] Díaz S, Lavorel S, De Bello F, Quétier F, Grigulis K, Robson TM 2007 Incorporating plant functional diversity effects in ecosystem service assessments. Proceedings of the National Academy of Sciences of the USA 104:20684–20689. 10.1073/pnas.0704716104 18093933 PMC2410063

[PLV147C13] Elton C 1958 The ecology of invasions by animal and plants, 4th edn London: Methuen , 181.

[PLV147C14] Evans C, Taylor DD 2011 New invader profile: Japanese chaff flower *Achyranthes japonica*. Wildland Weeds Summer/Fall:4–6.

[PLV147C15] Evans JA, Tranel PJ, Hager AG, Schutte B, Wu C, Chatham LA, Davis AS 2015 Managing the evolution of herbicide resistance. Pest Management Science 72:74–80. 10.1002/ps.4009 25809409 PMC5029781

[PLV147C16] Garnier E, Navas M-L 2012 A trait-based approach to comparative functional plant ecology: concepts, methods and applications for agroecology. A review. Agronomy for Sustainable Development 32:365–399. 10.1007/s13593-011-0036-y

[PLV147C17] Gibson DJ 2015 Methods in comparative plant population ecology, 2nd edn Oxford: Oxford University Press.

[PLV147C18] Gibson DJ, Schwartz LM 2014 Population dynamics of endangered *Iresine rhizomatosa* (Juda's bush). Grant Agreement No.: 13-026W Final Report to Illinois Department of Natural Resources http://opensiuc.lib.siu.edu/cgi/viewcontent.cgi?article=1004&context=pb_reports (20 March 2014).

[PLV147C19] Gibson DJ, Connolly J, Hartnett DC, Weidenhamer JD 1999 Designs for greenhouse studies of interactions between plants. Journal of Ecology 87:1–16. 10.1046/j.1365-2745.1999.00321.x

[PLV147C20] Goldberg DE, Landa K 1991 Competitive effect and response: hierarchies and correlated traits in the early stages of competition. Journal of Ecology 79:1013–1030. 10.2307/2261095

[PLV147C21] Guo P, Al-Khatib K 2003 Temperature effects on germination and growth of redroot pigweed (*Amaranthus retroflexus*), Palmer amaranth (*A. palmeri*), and common waterhemp (*A. rudis*). Weed Science 51:869–875. 10.1614/P2002-127

[PLV147C22] Hansen PK, Kristensen K, Willas J 2008 A weed suppressive index for spring barley (*Hordeum vulgare*) varieties. Weed Research 48:225–236. 10.1111/j.1365-3180.2008.00620.x

[PLV147C24] Hartzler RG, Buhler DD, Stoltenberg DE 1999 Emergence characteristics of four annual weed species. Weed Science 47:578–584.

[PLV147C25] Heap I 2014 Herbicide resistant weeds. In: Pimentel D, Peshin R, eds. Integrated pest management. New York, NY: Springer, 281–301.

[PLV147C26] Herman R, Miles C, Dungan L, Currie B, Ice P 1976 Soil survey of Jackson County, Illinois. Urbana Champaign, IL: United States Department of Agriculture, Soil Conservation Service and Forest Service, in cooperation with University of Illinois Agricultural Experimental Station.

[PLV147C27] Hock SM, Knezevic SZ, Martin AR, Lindquist JL 2006 Soybean row spacing and weed emergence time influence weed competitiveness and competitive indices. Weed Science 54:38–46. 10.1614/WS-05-011R.1

[PLV147C28] Holt JS 1995 Plant responses to light: a potential tool for weed management. Weed Science 43:474–482.

[PLV147C29] Horak MJ, Loughin TM 2000 Growth analysis of four *Amaranthus* species. Weed Science 48:347–355. 10.1614/0043-1745(2000)048[0347:GAOFAS]2.0.CO;2

[PLV147C31] [IDNR] Illinois Department of Energy and Natural Resources. 1994 The changing Illinois environment: critical trends. Summary Report and Volumes 1–7 Technical Report Springfield, IL: Illinois Department of Energy and Natural Resources.

[PLV147C33] Klingaman TE, Oliver LR 1994 Palmer amaranth (*Amaranthus palmeri*) interference in soybeans (*Glycine max*). Weed Science 42:523–527.

[PLV147C34] Kraft NJB, Godoy O, Levine JM 2015 Plant functional traits and the multidimensional nature of species coexistence. Proceedings of the National Academy of Sciences of the USA 112:797–802. 10.1073/pnas.1413650112 25561561 PMC4311865

[PLV147C35] Krueger-Mangold J, Sheley R, Engel R 2006 Can R*s predict invasion in semi-arid grasslands? Biological Invasions 8:1343–1354. 10.1007/s10530-005-0709-z

[PLV147C35a] Lehman C, Tilman D 2000 Biodiversity, stability, and productivity in competitive communities. The American Naturalist 156:534–552. 10.1890/06-1993.1 29587515

[PLV147C36] Maron J, Marler M 2007 Native plant diversity resists invasion at both low and high resource levels. Ecology 88:2651–2661. 10.1890/06-1993.1 18027767

[PLV147C37] McGill BJ, Enquist BJ, Weiher E, Westoby M 2006 Rebuilding community ecology from functional traits. Trends in Ecology and Evolution 21:178–185. 10.1016/j.tree.2006.02.002 16701083

[PLV147C38] Miller TE, Burns JH, Munguia P, Walters EL, Kneitel JM, Richards PM, Mouquet N, Buckley HL 2005 A critical review of twenty years’ use of the resource-ratio theory. The American Naturalist 165:439–448. 10.1086/428681 15791536

[PLV147C39] Mohlenbrock RH 2014 Vascular flora of Illinois: a field guide, 4th edn Carbondale, IL: Southern Illinois University Press.

[PLV147C40] Novoplansky A 1991 Developmental responses of *Portulaca* seedlings to conflicting spectral signals. Oecologia 88:138–140. 10.1007/BF00328414 28312742

[PLV147C41] Patterson DT 1985 Comparative ecophysiology of weeds and crops. In: Duke SO, ed. Weed physiology, Vol. 1 Boca Raton, FL: CRC Press, Inc.

[PLV147C42] Pimentel D, Zuniga R, Morrison D 2005 Update on the environmental and economic costs associated with alien-invasive species in the United States. Ecological Economics 52:273–288. 10.1016/j.ecolecon.2004.10.002

[PLV147C43] Sage RF, Sage TL, Pearcy RW, Borsch T 2007 The taxonomic distribution of C_4_ photosynthesis in Amaranthaceae *sensu stricto*. American Journal of Botany 94:1992–2003. 10.3732/ajb.94.12.1992 21636394

[PLV147C44] Sakai AK, Allendorf FW, Holt JS, Lodge DM, Molofsky J, With KA, Baughman S, Cabin RJ, Cohen JE, Ellstrand NC, McCauley DE, O'neil P, Parker IM, Thompson JN, Weller SG 2001 The population biology of invasive species. Annual Review of Ecology and Systematics 32:305–332. 10.1146/annurev.ecolsys.32.081501.114037

[PLV147C45] Schwartz LM 2014 Japanese chaff flower: a rising threat to southern Illinois. River to River Cooperative Weed Management Area http://rtrcwma.blogspot.com/2014/05/japanesechaff-flower-rising-threat-to.html (25 March 2015).

[PLV147C46] Schwartz LM 2015 A comparative study of the population dynamics of four Amaranthaceae species. PhD Dissertation, Southern Illinois University, Carbondale, IL.

[PLV147C47] Schwartz LM, Smith KM, Evans C, Gage KL, Gibson DJ, Young BG 2015 Fact sheet: ecology and control of Japanese chaff flower [*Achyranthes japonica* (Miq.) Nakai]. River to River Cooperative Weed Management Area http://www.rtrcwma.org/Chaff_FactSheet.pdf (25 March 2015).

[PLV147C48] Schwartz LM, Gibson DJ, Young BG 2016 Life history of *Achyranthes japonica* (Amaranthaceae): an invasive species in southern Illinois. Journal of the Torrey Botanical Society 143: in press.

[PLV147C49] Simberloff D, Schmitz DC, Brown TC 1997 Strangers in paradise: impact and management of non-indigenous species in Florida. Washington, DC: Island Press.

[PLV147C50] Smith H 2000 Phytochromes and light signal perception by plants—an emerging synthesis. Nature 407:585–591. 10.1038/35036500 11034200

[PLV147C51] Smith KM 2013 Invasion and management of Achyranthes japonica in a southern Illinois wetland. Master's Thesis, Southern Illinois University, Carbondale, IL http://opensiuc.lib.siu.edu/theses/1337 (25 March 2015).

[PLV147C52] Steckel LE, Sprague CL 2004 Common waterhemp (*Amaranthus rudis*) interference in corn. Weed Science 52:359–364. 10.1614/WS-03-066R1

[PLV147C53] Steckel LE, Sprague CL, Stoller EW, Wax LM 2004 Temperature effects on germination of nine *Amaranthus* species. Weed Science 52:217–221. 10.1614/WS-03-012R

[PLV147C54] Steckel LE, Main CL, Ellis AT, Mueller TC 2008 Palmer amaranth (*Amaranthus palmeri*) in Tennessee has low level glyphosate resistance. Weed Technology 22:119–123. 10.1614/WT-07-061.1

[PLV147C55] Tilman D 1982 Resource competition and community structure. Princeton, NJ: Princeton University Press. 7162524

[PLV147C56] Tilman D 1985 The resource-ratio hypothesis of plant succession. The American Naturalist 125:827–852. 10.1086/284382

[PLV147C57] Tilman D 1987 On the meaning of competition and the mechanisms of competitive superiority. Functional Ecology 1:304–315. 10.2307/2389785

[PLV147C58] Tilman D 1988 Plant strategies and the dynamics and structure of plant communities. Princeton, NJ: Princeton University Press.

[PLV147C59] Tilman D 1997 Mechanisms of plant competition. In: Crawley M, ed. Plant ecology, 2nd edn Oxford: Blackwell Science, 239–261.

[PLV147C62] Trucco F, Tranel PJ 2011 Amaranthus. In: Kole C, ed. Wild crop relatives: genomic and breeding resources. Berlin: Springer, 11–21.

[PLV147C63] Vencill WK, Grey TL, Culpepper AS, Gaines TA, Westra P 2008 Herbicide-resistance in the Amaranthaceae. Journal of Plant Diseases and Protection XXI (Special Issue):41–44.

[PLV147C64] Violle C, Garnier E, Lecoeur J, Roumet C, Podeur C, Blanchard A, Navas M 2009 Competition, traits and resource depletion in plant communities. Community Ecology 160:747–755. 10.1007/s00442-009-1333-x 19352713

[PLV147C65] Wang P, Stieglitz T, Zhou DW, Cahill JF Jr 2010 Are competitive effect and response two sides of the same coin, or fundamentally different? Functional Ecology 24:196–207. 10.1111/j.1365-2435.2009.01612.x

[PLV147C66] Ward SM, Webster TM, Steckel LE 2013 Palmer Amaranth (*Amaranthus palmeri*): a review. Weed Technology 27:12–27. 10.1614/WT-D-12-00113.1

[PLV147C67] Westbrooks RG 2004 New approaches for early detection and rapid response to invasive plants in the United States. Weed Technology 18:1468–1471. 10.1614/0890-037X(2004)018[1468:NAFEDA]2.0.CO;2

[PLV147C68] Yang F, Huang S, Gao R, Liu W, Yong T, Wang X, Wu X, Yang W 2014 Growth of soybean seedlings in relay strip intercropping systems in relation to light quantity and red:far-red ratio. Field Crops Research 155:245–253. 10.1016/j.fcr.2013.08.011

[PLV147C69] Zhang S, Lamb EG 2012 Plant competitive ability and the transitivity of competitive hierarchies change with plant age. Plant Ecology 213:15–23. 10.1007/s11258-011-0002-4

[PLV147C70] Zimdahl RL 2004 Weed-crop competition: a review. Ames, IA: Blackwell Publishing.

