# Supplementary material for: Do plant traits predict the competitive abilities of closely related species?
Source: AoB Plants. 2015 Dec 31;8:plv147. doi: 10.1093/aobpla/plv147 (PMC4719039; doi:10.1093/aobpla/plv147)
Supplement: Additional Information [file supp_8_plv147_index.html]

Do plant traits predict the competitive abilities of closely related species? — Additional Information 

# Do plant traits predict the competitive abilities of closely related species?

## Additional Information

Additional Information

- Additional Information - Docx file
