# Supplementary material for: Do plant traits predict the competitive abilities of closely related species?
Source: AoB Plants. 2015 Dec 31;8:plv147. doi: 10.1093/aobpla/plv147 (PMC4719039; doi:10.1093/aobpla/plv147)
Supplement: Additional Information [file supp_plv147_plv147supp.docx]

**Supporting Information**

**File 1. Figure.** Mean (± se) soil moisture for a) trial 2 2013, b) trial 1 2013, c) trial 2 2014, and d) trial 1 2014. Red lines are indicative of daily average soil moisture. Mean values with the same letters are not significantly different at α = 0.05 within a species.

**File 2. Figure.** Mean (± se) light intensity at the soil surface for a) trial 2 2013, b) trial 1 2013, c) trial 2 2014, and d) trial 1 2014.
